# A global population assessment of the Chinstrap penguin (*Pygoscelis antarctica*)

**DOI:** 10.1038/s41598-020-76479-3

**Published:** 2020-11-10

**Authors:** Noah Strycker, Michael Wethington, Alex Borowicz, Steve Forrest, Chandi Witharana, Tom Hart, Heather J. Lynch

**Affiliations:** 1 School of Marine and Atmospheric Sciences, Stony Brook University, 145 Endeavour Hall, Stony Brook, NY 11794 USA; 2 Department of Ecology and Evolution, Stony Brook University, Stony Brook, NY USA; 3 Department of Natural Resources and the Environment, University of Connecticut, Storrs, CT USA; 4 Department of Zoology, University of Oxford, Oxford, UK; 5 Institute for Advanced Computational Science, Stony Brook University, Stony Brook, NY USA

**Keywords:** Ecology, Ocean sciences

## Abstract

Using satellite imagery, drone imagery, and ground counts, we have assembled the first comprehensive global population assessment of Chinstrap penguins (*Pygoscelis antarctica*) at 3.42 (95th-percentile CI: [2.98, 4.00]) million breeding pairs across 375 extant colonies. Twenty-three previously known Chinstrap penguin colonies are found to be absent or extirpated. We identify five new colonies, and 21 additional colonies previously unreported and likely missed by previous surveys. Limited or imprecise historical data prohibit our assessment of population change at 35% of all Chinstrap penguin colonies. Of colonies for which a comparison can be made to historical counts in the 1980s, 45% have probably or certainly declined and 18% have probably or certainly increased. Several large colonies in the South Sandwich Islands, where conditions apparently remain favorable for Chinstrap penguins, cannot be assessed against a historical benchmark. Our population assessment provides a detailed baseline for quantifying future changes in Chinstrap penguin abundance, sheds new light on the environmental drivers of Chinstrap penguin population dynamics in Antarctica, and contributes to ongoing monitoring and conservation efforts at a time of climate change and concerns over declining krill abundance in the Southern Ocean.

## Introduction

Chinstrap penguins (*Pygoscelis antarctica*) are abundant in Antarctica, with past estimates ranging from 3–8 million breeding pairs, and are considered a species of “least concern” by BirdLife International^1^, but the population dynamics of this species are not well understood and several studies have highlighted significant declines at monitored sites^2–6^. Because Chinstrap penguins nest in remote and rugged areas, on-the-ground census work is difficult, expensive, and sporadic. As a result, documentation of global Chinstrap penguin abundance and distribution has, to date, been unavoidably incomplete, and many regions where Chinstrap penguins breed have not been visited or surveyed since the early 1980s. Without an accurate count, it is impossible to form a complete picture of the species’ distribution and assess its population dynamics over time.

The literature on Chinstrap penguin abundance and population trends suggests that Chinstrap penguin numbers in Antarctica increased during the decades leading up to the 1970s^7,8^ and subsequently declined. Since the 1980s, breeding populations in some areas have been reported to have declined by > 50%^2–6,8,9^, with notable exceptions in the South Sandwich Islands, at South Georgia Island, and in a few sites near the southern extent of the Chinstrap penguin’s range^4,10^. Because of their dependence on krill (*Euphausia* spp.) and fish (*Pleuragramma antarctica*), Chinstrap penguins can be viewed as “marine sentinels” for quantifying environmental change in the Southern Ocean^11^. Despite considerable interest in the dynamics of this species as a window into the Southern Ocean ecosystem^12^, reliable population estimates for Chinstrap penguins are lacking, both globally and regionally. Updated counts would improve estimates of krill consumption by Chinstrap penguins and benefit spatial planning efforts, including the design of protected areas.

Accurate, longitudinal data are also needed to test hypotheses attempting to explain why both Chinstrap and Adélie (*Pygoscelis adeliae*) penguin populations around the Western Antarctic Peninsula have declined for the past half century while Gentoo penguins (*Pygoscelis papua*) have increased, in many cases at colonies where the species nest side by side (e.g.^8,13,14^). Popular hypotheses suggest that penguin populations are driven by krill availability, but krill biomass is broadly affected by climate change, krill fishing, and the recovery of whale and seal populations—the collective effects of which are difficult to disentangle from the perspective of penguin dynamics^8,15–17^. Other potential impacts, including tourism, extreme weather events, and disease outbreaks, may be important at local scales or for short periods of time, but are less compelling explanations for the widespread changes observed across the Antarctic penguins^3,18,19^.

The purpose of this paper is to address two research questions: (1) What is the global population and distribution of Chinstrap penguins? and (2) How does updated information on Chinstrap penguin abundance and distribution support or refute current hypotheses of penguin population dynamics around the Western Antarctic Peninsula? This paper provides a status report on our efforts to assemble all the available information on Chinstrap penguin distribution, abundance, and population trends over the past 40 years. We have used published data, additional unpublished data from our own recent field surveys, and estimates derived from high-resolution [0.31–3.0 m/pixel] satellite imagery obtained from Maxar, Planet, and Google Earth, as well as medium-resolution [30.0 m/pixel] Landsat imagery and unmanned aerial system (UAS) imagery. While unavoidably incomplete, this report provides the most comprehensive catalog to date of Chinstrap penguin colonies, their exact locations, and their population trends (see Supplementary Information), and identifies priority areas for future surveys. Finally, we discuss how these results fit into the current debate surrounding drivers of penguin population trends in the Antarctic Peninsula region.

## Results

We estimate the global population of Chinstrap penguins at 3.42 (95th-percentile CI: [2.98, 4.00]) million breeding pairs (Table 1) in 375 extant breeding sites, not including recent extirpations. All survey details, updated population estimates, historical benchmarks, and estimates of population change are provided in the Supplementary Information.

**Table 1:** Estimated abundance (in breeding pairs) by CCAMLR subarea.

Unit	Abundance (count)	Abundance (model)	95th percentile CI (model)
**Subarea 48.1**	1,099,260	1,108,348	909,041–1,324,192
*Small-Scale Management Unit*			
Antarctic Peninsula Elephant Island	253,243	257,019	186,619–329,230
Antarctic Peninsula Drake Passage East	231,369	234,644	165,337–305,717
Antarctic Peninsula Drake Passage West	91,465	92,071	71,534–114,160
Antarctic Peninsula Bransfield Strait East	78,806	78,861	58,764–98,021
Antarctic Peninsula Bransfield Strait West	424,862	426,553	255,682–604,100
Antarctic Peninsula West	19,501	19,643	15,308–24,452
Antarctic Peninsula East	14	14	13–15
**Subarea 48.2–South Orkney Islands**	960,451	979,011	694,791–1,260,452
*Small-Scale Management Unit*			
South Orkney North East	201,948	202,929	147,126–258,740
South Orkney South East	756,503	768,519	513,524–1,058,744
South Orkney West	2,000	2,056	353–3,858
**Subarea 48.3–South Georgia Island**	13,434	13,500	10,005–16,915
**Subarea 48.4–South Sandwich Islands**	1,349,500	1,366,553	970,345–1,726,931
**Subarea 48.6–Bouvet Island**	60	60	55–65
**Subarea 88.1–Balleny Islands**	124	124	49–203
Total	3,422,829	3,471,670	2,981,764–3,997,372

**Figure 1:**
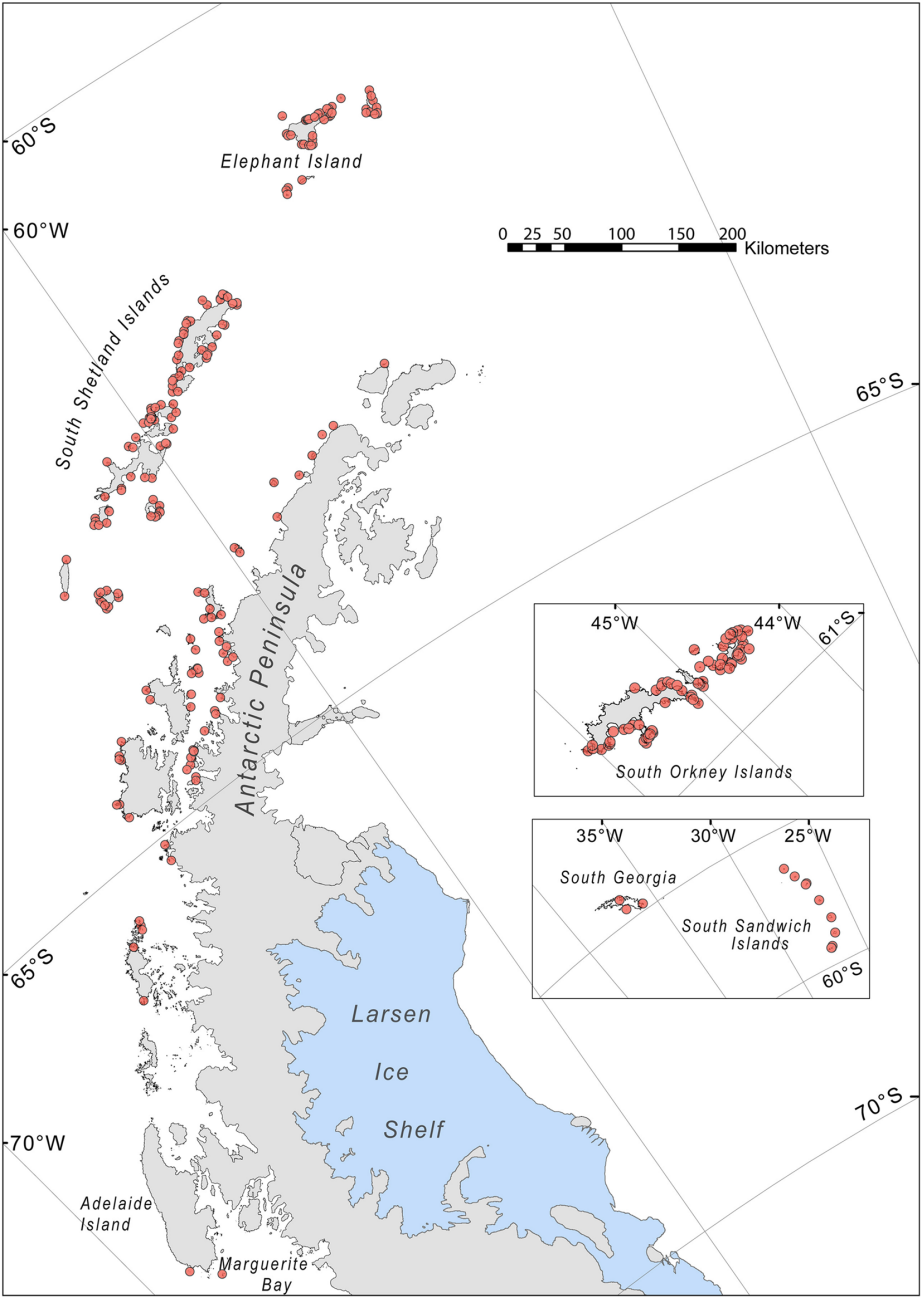
Map of extant Chinstrap penguin colonies on the Antarctic Peninsula and nearby subantarctic islands. Figure created with ArcMap version 10.6.1 and Adobe Illustrator 2020 version 24.2.1.

**Figure 2:**
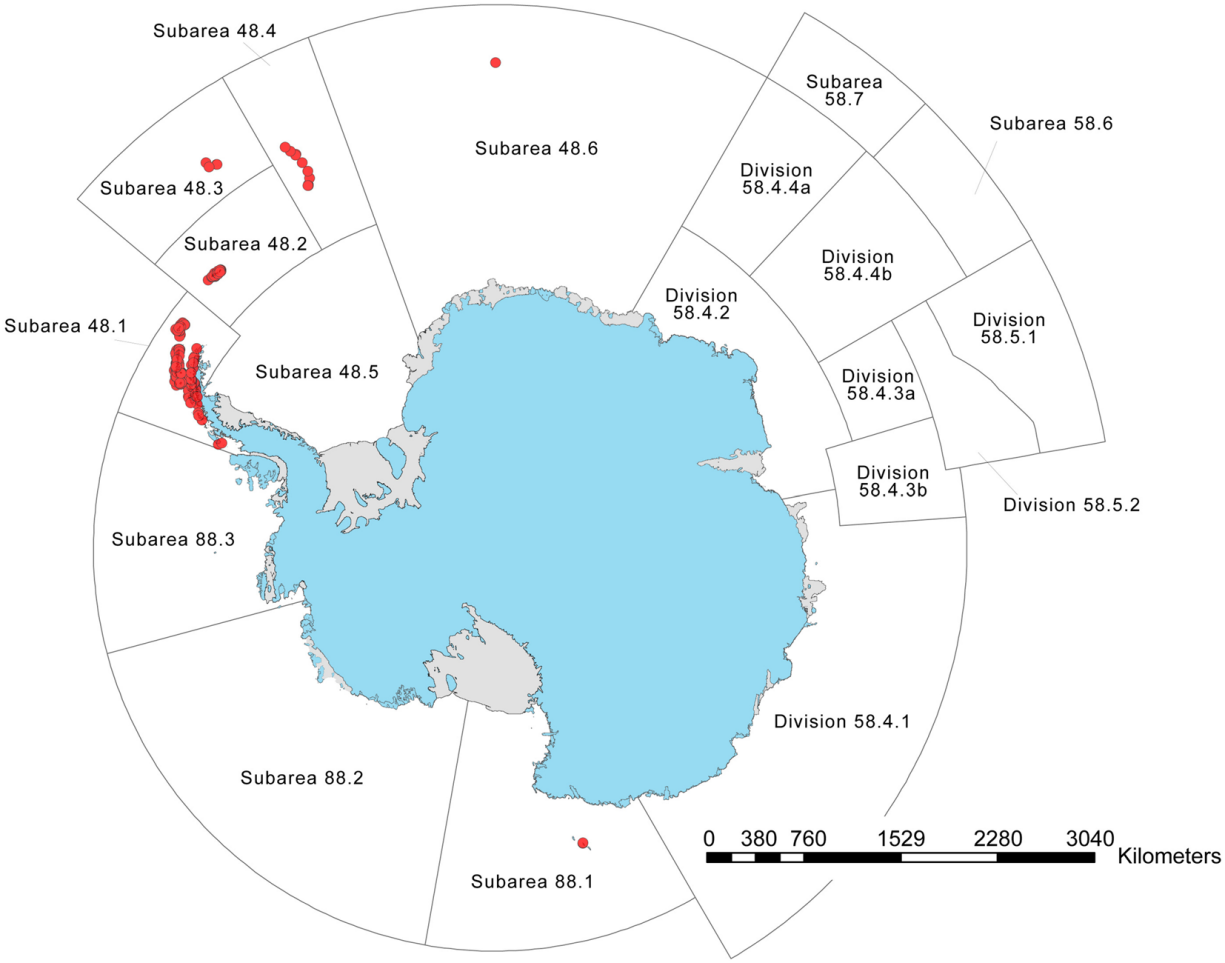
Map of all extant Chinstrap penguin colonies by CCAMLR subarea. Figure created with ArcMap version 10.6.1 and Adobe Illustrator 2020 version 24.2.1.

Most Chinstrap penguin colonies are in the southwest Atlantic sector of the Southern Ocean, which includes the Antarctic Peninsula and associated islands, including the South Orkney Islands, South Sandwich Islands, and South Georgia Island (Fig. 1). The southernmost Antarctic Peninsula colonies are located on the north side of Marguerite Bay, at a latitude of approximately 67.8′ S. Globally, the Chinstrap penguin’s range also includes small colonies on Bouvet Island and in the Balleny Islands (Fig. 2). Of 398 total sites, we were able to verify the locations of 364 from satellite imagery (Table 2). Colonies were present, or presumed present, at 332 sites, with an additional 42 colonies unable to be assessed with available literature and imagery. This total includes 26 previously unreported colonies that, which the exception of several small colonies in the far south, were likely present but overlooked by previous surveys. There were 23 colonies that were either confirmed as having no breeding Chinstrap penguins or where we presume, but have not confirmed, absence—all of which represent potential extirpations. For 260 sites with updated abundance estimates, 43.8% had the highest level of precision (N1, ± 5% accuracy) and 42.3% had the lowest (N5, nearest order of magnitude), following accepted standards (e.g.^20,21^). While we did identify several previously unreported colonies, the identification of very small colonies (< 10 breeding pairs) is difficult, and it is likely that additional, small colonies will be discovered over time. These colonies do not significantly affect global totals.

**Table 2:** Colony status, size, and survey precision for updated estimates (*N* = 398 sites).

	Number of sites	% of total
Location verified by satellite imagery	364	91.5
**Current colony status**		
Present	265	66.6
Presumed present	68	17.1
Absent	21	5.3
Presumed absent	2	0.5
Unknown	42	10.6
**Colony size (most recent estimate)**		
1–10	19	4.8
11–100	51	12.8
101–1000	109	27.5
1001–10,000	142	35.8
10,001–100,000	40	10.1
100,000 +	6	1.5
**Precision of current estimate (n = 283)**		
Nest count		
N1 (± 5% accuracy)	114	40.3
N2 (± 10%)	20	7.1
N3 (± 10–15%)	8	2.8
N4 (± 25–50%)	5	1.8
N5 (nearest order of magnitude)	119	42.0
Chick count (C1; ± 5%)	14	4.9
Adult count (A4; ± 25–50%)	3	1.1

Using the best current estimates of abundance, mean colony size for 367 colonies was 9327 breeding pairs (SD = 40,861) (median = 1100), excluding the 23 extirpated sites and eight sites with no current abundance information. A total of 19.1% of these colonies fell between 1 and 100 breeding pairs, 68.4% fell between 101 and 10,000 breeding pairs, and 12.5% had more than 10,000 breeding pairs.

To summarize the distribution of Chinstrap penguins in a management context, we use regions previously defined by the Commission for the Conservation of Antarctic Marine Living Resources (CCAMLR). Within CCAMLR subarea 48.1, which includes the Antarctic Peninsula and adjacent islands, 75.2% of breeding Chinstrap penguins were located in the South Shetland Islands and 23.0% in the Elephant Island region; smaller numbers were in the Central-West, Northwest, and Southwest Antarctic Peninsula areas (Fig. 1). Across all CCAMLR subareas (Fig. 2), Chinstrap penguin breeding colonies were distributed as follows: 39.4% in subarea 48.4 (South Sandwich Islands), 32.1% in subarea 48.1 (Antarctic Peninsula and adjacent islands), 28.1% in subarea 48.2 (South Orkney Islands), and 0.4% in subarea 48.3 (South Georgia Island), with smaller populations in subareas 48.6 (Bouvet Island) and 88.1 (Balleny Islands).

**Figure 3:**
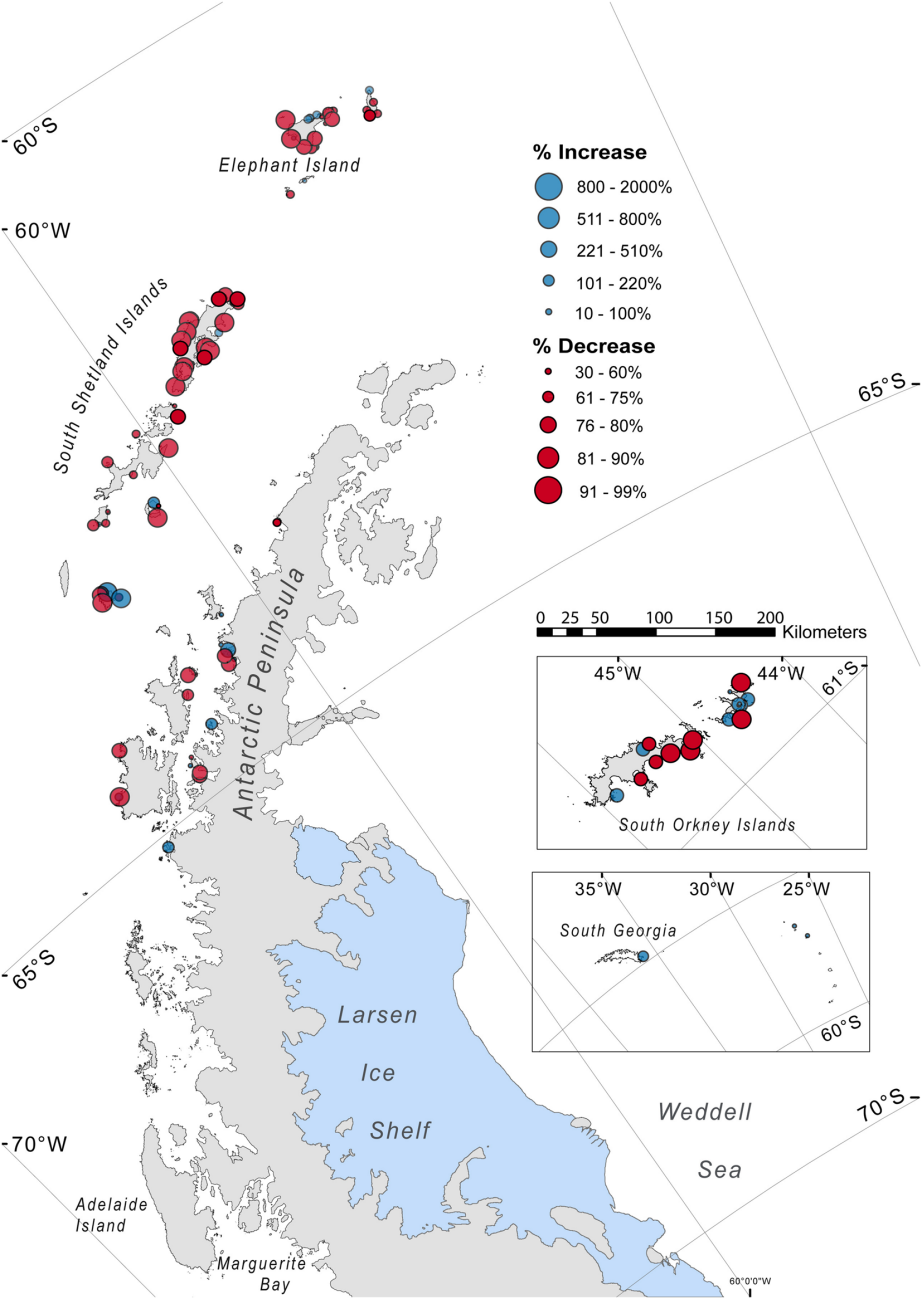
Chinstrap penguin colonies for which a historic benchmark is available, with significant population changes since the 1980s. Figure created with ArcMap version 10.6.1 and Adobe Illustrator 2020 version 24.2.1.

Population changes are summarized by region and Small-Scale Management Unit in Table 3. Overall, 34.7% of colonies could not be assessed against a historical benchmark. Of those colonies for which a comparison could be made to counts in the 1980s, 40.4% have declined, 16.2% have increased, 24.6% have not changed significantly, 10.0% represent previously unrecognized colonies, and 8.8% have been extirpated. However, this picture changes when assessing total population rather than number of colonies. Of the same sample, 15.3% of the total population are in colonies that have declined or probably declined and 25.1% in colonies that have increased or probably increased, with 57.6% of the population not changing significantly and 2.0% representing previously unrecognized colonies. This is due to the fact that more than one-third of the global Chinstrap penguin population is concentrated in a few large colonies in the South Sandwich Islands, where the species is apparently stable^4^. For colonies that could be assessed against a historic benchmark, most are declining on the Western Antarctic Peninsula (Fig. 3), while trends are mixed elsewhere. Many colonies in the eastern part of the Chinstrap penguin’s range could not be assessed because of limited historic data; these are not shown in the figure.

**Table 3:** Change in Chinstrap penguin abundance by CCAMLR subarea.

	Increase		Decrease		New colony		Extirpated		No change		Unknown	
	% Cols	% Total	% Cols	% Total	% Cols	% Total	% Cols	% Total	% Cols	% Total	% Cols	% Total
Overall	10.6	14.5	26.4	8.8	6.5	1.1	5.8	0.0	16.1	33.3	34.7	42.2
Subarea 48.1	9.1	13.6	34.9	24.8	7.6	3.4	6.5	0.0	13.5	12.6	28.4	45.6
APEI	13.0	37.0	50.0	43.7	5.6	0.3	1.9	0.0	14.8	11.1	14.8	8.0
APDPE	2.5	3.1	37.5	12.4	10.0	2.4	5.0	0.0	7.5	10.5	37.5	71.6
APDPW	3.7	21.9	22.2	15.6	22.2	30.1	7.4	0.0	14.8	11.1	29.6	21.3
APBSE	6.9	5.1	62.1	22.1	3.4	3.8	0.0	0.0	6.9	12.8	20.7	56.1
APBSW	12.8	5.0	25.6	23.2	2.6	0.1	9.0	0.0	9.0	14.4	41.0	57.4
APE	0.0	0.0	0.0	0.0	0.0	0.0	0.0	0.0	0.0	0.0	100	100
APW	8.2	19.0	20.4	15.3	10.2	0.0	12.2	0.0	26.5	22.7	22.4	42.9
Subarea 48.2	12.9	6.4	8.6	3.1	5.4	0.2	3.2	0.0	25.8	20.2	44.1	70.1
SONE	17.9	12.3	7.1	0.6	7.1	0.4	3.6	0.0	25.0	35.9	39.3	50.8
SOSE	10.9	4.9	9.4	3.8	4.7	0.1	3.1	0.0	25.0	15.8	46.9	75.4
SOW	0.0	0.0	0.0	0.0	0.0	0.0	0.0	0.0	100	100	0.0	0.0
Subarea 48.3	6.3	42.5	0.0	0.0	0.0	0.0	12.5	0.0	0.0	0.0	81.3	57.5
Subarea 48.4	20.0	20.7	0.0	0.0	0.0	0.0	0.0	0.0	30.0	59.7	50.0	19.5
Subarea 48.6	0.0	0.0	50.0	83.3	0.0	0.0	0.0	0.0	0.0	0.0	50.0	16.7
Subarea 88.1	100	100	0.0	0.0	0.0	0.0	0.0	0.0	0.0	0.0	0.0	0.0

% colonies = number of colonies divided by total colonies in each Small-Scale Management Unit or subarea; % total = breeding pairs in the colonies within each category divided by total number of pairs in each Small-Scale Management Unit or subarea.

*APEI* Antarctic Peninsula Elephant Island, *APDPE* Antarctic Peninsula Drake Passage East, *APDPW* Antarctic Peninsula Drake Passage West, *APBSE* Antarctic Peninsula Bransfield Strait East, *APBSW* Antarctic Peninsula Bransfield Strait West, *APE* Antarctic Peninsula East, *APW* Antarctic Peninsula West, *SONE* South Orkney North East, *SOSE* South Orkney South East, *SOW* South Orkney West, *Subarea 48.3* South Georgia Island, *Subarea 48.4* South Sandwich Islands, *Subarea 48.6* Bouvet Island, *Subarea 88.1* Balleny Islands.

## Discussion

We estimate Chinstrap penguin abundance at 3.42 million breeding pairs. This estimate is broadly consistent with the BirdLife International total of 8 million mature individuals^1^. The BirdLife estimate required an update because it did not incorporate recent compilations of abundance, include full spatial coverage, or provide a robust treatment of uncertainty. Thus, this study provides a rigorous baseline against which past and future trends can be assessed. While substantively complete, our efforts to calculate a global Chinstrap penguin assessment remain a work in progress, as additional satellite imagery and field expeditions over the coming years will undoubtedly fill gaps in our understanding of current abundance and distribution.

To place our findings on the current abundance and distribution of Chinstrap penguins in context, we briefly summarize the various hypotheses put forth to explain why Chinstrap penguin populations have fluctuated dramatically since the very earliest days of monitoring efforts and provide some suggestions for future research to address gaps in our knowledge uncovered by this population assessment.

### Krill vs. climate: 50 years of debate

Hypotheses concerning population changes of *Pygoscelis* spp. penguins on the relatively well-studied Western Antarctic Peninsula have evolved over the past half century. In the 1970s, Chinstrap penguin numbers were understood to be increasing^22^. Sources from that period attributed the change to a krill surplus caused by the commercial decimation of Southern Ocean whale populations earlier in the century^20,22^. Competition with whales, and possibly Antarctic fur seals, remains a parsimonious hypothesis for Chinstrap penguin population fluctuations; as whale and fur seal numbers have rebounded in recent decades^23^ Chinstrap penguins have simultaneously begun to decline. Notably, however, sympatric populations of the other pygoscelid penguins—Adélie and Gentoo—have not behaved similarly. Fraser et al.^15^ observed that while Chinstrap penguin populations had increased through the 1980s, Adélie penguins had not. Because both species are krill specialists, competition alone could not explain the disparity. Their “sea ice hypothesis” proposed that Chinstrap penguins, as a pagophobic species, had benefitted from a long-term decline in sea ice cover. Although this prediction did not fit the subsequent drop in Chinstrap penguin numbers on the Western Antarctic Peninsula, where sea ice decline has continued, it helped shape later discussions relating life history characteristics to penguin population change in the context of environmental impacts.

Reviewing both Chinstrap and Adélie penguin populations, Trivelpiece et al.^8^ formulated an inclusive hypothesis relating penguin populations to krill biomass. Competition from rebounding whale and fur seal populations, the effects of climate change on sea ice used by larval krill, and the development of a pelagic krill-trawling fishery were each proposed to exert downward pressure on total krill biomass, with cascading effects on penguin populations. This overall “krill hypothesis” remains a reasonable model for the decline of Chinstrap penguins on the Western Antarctic Peninsula since the 1980s, but it cannot distinguish which of these broad impacts is most significant. It also does not address the rapid increase in sympatric Gentoo penguin populations during the same period. Although Gentoo penguins have a somewhat more flexible diet, they also survive predominantly on krill during the Antarctic summer and often nest in the same colonies as Chinstrap and Adélie penguins^24,25^.

While competition from whales and fur seals cannot be ruled out as a contributing factor in the rapid decline of some krill-dependent species, most of the focus has been on climate change and krill fishing as the two most likely principal drivers underpinning the observed changes in pygoscelid composition. Scientists have increasingly emphasized the impacts of rising temperatures and retreating sea ice due to global climate change (e.g.^26,27^), especially where temperature increases have been disproportionately high on the Western Antarctic Peninsula. Warmer temperatures in this region are well documented; for instance, Bromwich et al.^28^ found a 2.4 °C increase in Western Antarctic temperatures from 1958 to 2012. Extreme weather events, including record high air temperatures, are also evidently becoming more frequent^29^. Climate change, by influencing sea ice dynamics and marine environmental conditions, likely affects the biomass and distribution of krill in the Southern Ocean^30^ and, in turn, penguin populations^8^.

Krill fishing may play a significant role in Chinstrap penguin population dynamics, especially at local scales^17^. Multiple studies have documented overlap between commercial krill trawls and penguin foraging ranges^16,31,32^. Because they make marginally longer foraging trips than Adélie or Gentoo penguins, Chinstrap penguins could have higher exposure to fishing interference during the breeding season. Watters et al.^32^ predicted that today’s “precautionary” limits on krill fishing would affect penguin breeding success with a similar magnitude to climate change, if seasonal local harvest rates exceed a threshold of 0.1. This is especially pertinent to Chinstrap penguin colonies adjacent to krill-fishing hotspots, though continued declines in areas currently experiencing less intense krill trawling (e.g. Elephant Island^6^) suggest that krill fishing does not fully explain the widespread Chinstrap penguin population changes identified by our assessment.

### A renewed focus on the overwinter  period

If breeding success is the primary driver of population change in pygoscelid penguins, then one might expect clear distinctions between Chinstrap, Adélie, and Gentoo penguin productivity throughout the Western Antarctic Peninsula over the past half century. However, several decades-long investigations of these penguins nesting together, in mixed colonies, have reported no significant difference in chicks fledged (e.g.^5,13^). As previously argued by Hinke et al.^13^, these data strongly indicate that contemporary Chinstrap penguin declines are largely driven by factors in the non-breeding portion of the year rather than by summer production.

All three *Pygoscelis* spp. are central-place foragers at their summer colonies with heavily overlapping ranges^16^. After the nesting season, though, each occupies a very different niche and geographic area. Chinstrap penguins migrate the farthest, swimming up to 4500 km away from their colonies to overwinter in a latitude near 60° south in the Southern Ocean; those from the Western Antarctic Peninsula mostly travel westward^33,34^. Adélie penguins from the northern Antarctic Peninsula migrate east to overwinter in the Weddell Sea pack ice zone^33^. Gentoo penguins, meanwhile, stay close to their Antarctic Peninsula breeding colonies throughout the winter^16^.

If austral summer conditions are nominally similar for all three sympatrically breeding *Pygoscelis* spp., could we be observing a mismatch between the timing or location of winter resources and overwinter foraging behavior? Such trophic mismatches have been an area of concern for other bird species, and long-distance migrant birds have declined at a faster rate than resident species in response to global climate change (e.g.^35,36^, see also^37^). This has generally been explained as a disconnect between inflexible migratory instincts and rapidly changing environmental conditions in one part of the birds’ range, a concept known as “decoupling”^38^. If the Southern Ocean ecosystem is changing faster than migratory penguins can adapt, then long-distance migrants—Chinstrap and Adélie penguins—could be disproportionately affected.

One area of concern salient to understanding Chinstrap penguin population declines is that krill biomass may be shifting. In a wide analysis of krill trawl data, Atkinson et al.^39^ found that krill have become more concentrated near Antarctic shelves, krill densities have declined near the species’ northern limit, and the range of Antarctic krill has contracted south by more than 400 km in the past 90 years. Winter tracking data show Chinstrap penguins foraging in a narrow latitude along the northern edge of known krill distribution, in a zone vulnerable to such a shift^34^. If the winter range of these penguins is out of sync with krill concentrations, and if Chinstrap penguins depend on krill year-round, then a decoupling hypothesis specific to the nonbreeding season could predict the decline of Chinstrap penguin populations while allowing for an increase in Gentoo penguins, and would be consistent with the similar breeding productivity of all three pygoscelid species. This perspective on the importance of Chinstrap penguin winter foraging is inspired by recent tracking studies, and suggests that continued research on the overwinter activities of the three *Pygoscelis* spp. penguins (regarding both foraging areas and diet) is key to understanding the extent to which changes in population reflect summer or winter conditions.

### Moving forward

While recent surveys in the Elephant Island and Low Island region have filled in some critical gaps for Chinstrap penguins^6^, our assessment has identified additional areas that should be considered priorities for future surveys. The South Orkney Islands contain several very large Chinstrap penguin colonies that should be resurveyed, particularly in the vicinity of Sandefjord Bay and Monroe Island as well as on Saddle Island. The South Sandwich Islands contain some of the largest and most poorly surveyed Chinstrap penguin colonies globally, though recent UAS imagery has been collected that may soon provide updated population counts for several of these sites. The Aitcho Islands group just west of Robert Island hosts a number of previously unreported Chinstrap penguin colonies that were first identified in Landsat satellite imagery and have since been confirmed using ground surveys and UAS imagery. Though none of these colonies is particularly large, there remain several Chinstrap penguin colonies which have never been surveyed. A complete exploration of this area is both logistically feasible and necessary. While we were recently able to census Cape Wallace on Low Island, we were unable to survey the similarly sized Cape Garry, which is likely to be one of the largest Chinstrap penguin colonies outside the South Sandwich Islands despite probable declines. Finally, as an aid to future surveys by satellite imagery, we have provided in the Supplementary Information shapefiles representing the bounding boxes for 139 colonies investigated using satellite imagery so these colonies can be easily relocated.

## Methods

In this population assessment, we refer to a group of nesting Chinstrap penguins (with a minimum size of one breeding pair) as a “colony,” and the location of snow and ice-free terrain where such a colony may be located as a breeding “site.” The distinction is important because sites are permanent and exist regardless of whether penguins colonize them. Most actual and potential breeding sites for Chinstrap penguins in Antarctica have been previously defined^42^; because of natal philopatry, each Chinstrap penguin breeding population is considered separate from other colonies at adjacent sites, although weak population structure implies some movement over generations^40,41^. Where we have subdivided sites or grouped sites relative to previous accounts, we have noted those distinctions in the database accompanying this report in the Supplementary Information.

Site names for this assessment were based on previous compilations; in some cases, names have been updated to reflect what is currently available through the Mapping Application for Penguin Populations and Projected Dynamics^42^. Colony locations have been cross-checked against available satellite imagery. A large number of sites have had their locations updated to reflect the precise location of the Chinstrap penguin colony. Subareas for breeding populations, as well as Small-Scale Management Units within subarea 48.1, followed CCAMLR definitions. In our compilation of Chinstrap penguin abundance, we prioritized census data from direct methods (ground counting of individual occupied nests or chicks, or counts based on UAS imagery). Where no updated count was available, we used satellite imagery to estimate the area of guano coverage at the colony, similar to the approach used by Lynch and LaRue^43^ for Adélie penguins, prioritizing high-resolution commercial imagery and using Planet or Landsat imagery only when no cloud-free high-resolution imagery was available. In six cases, we were able to confirm continued occupation of a site using satellite imagery, but were unable to estimate abundance, either because the guano signature was too diffuse or because a portion of the colony was obscured by clouds. In about one third of all sites, we were unable to update the abundance estimate for known colonies. Instead, for the purposes of estimating regional or global population totals, we used the most recently available estimate, noting that in most cases the older abundance estimates were from the 1980s. Insufficiently surveyed coastlines in Chinstrap penguin-dominated areas were visually searched in high-resolution satellite imagery; in this manner, we discovered a few new or previously unreported colonies.

On satellite images, Chinstrap penguin colonies were identified by the spatial and spectral characteristics of their guano^43^ and the manual delineation of guano was done based on experience in concert with historic maps of guano extent. Different species of penguins may sometimes be differentiated in high-resolution imagery^43^, but identifying Chinstrap penguin colonies at mixed-species sites required knowledge of the site or multiple images in which species could be distinguished based on breeding phenology. For that reason, we did not attempt to estimate Chinstrap penguin abundance using satellite imagery at mixed-species sites. Our determination as to the current status of a colony at a site followed the logic tree illustrated in Supplemental Figure S1. For colonies that we were unable to update, we considered all large colonies (> 499 breeding pairs at last census) to be “presumed present” whereas smaller colonies were designated as “unknown”.

Unlike Adélie penguins^43^, we do not yet have enough coincident satellite and ground count data to construct a rigorous statistical model for Chinstrap penguin density. In 10 instances, a ground count and satellite image coincided within a seven-year period, and we used these counts to estimate a very crude nesting density of 0.5 nests/m^2^. We have used this nesting density to convert the area of guano in satellite imagery to an estimate of the breeding population. Recognizing uncertainty of nesting density (beyond the uncertainty associated with guano area itself), we have designated all such population estimates as being in the lowest precision category (accuracy = 5, correct to the “nearest order of magnitude”). For these sites only, counts were rounded to the nearest hundred (< 1000), nearest thousand (< 100,000), or nearest hundred thousand.

In communicating the precision of each population estimate, we have followed the tradition established by previous accounts (e.g.^9,20,21,43^) by binning count accuracy on a 5-point scale (see Supplementary Information). Recognizing that our uncertainty on current abundance reflects both the uncertainty of the original survey and the time elapsed since the most recent survey of a colony, we have downgraded the precision of counts older than 2015 by either one step (e.g., from accuracy = 2 to accuracy = 3) for counts from 2005–2014, by two steps for counts from 1995–2004, and by three steps for counts from 1985–1994, noting that the accuracy code saturates at 5. Doing so allows us to most accurately communicate the uncertainty of our estimate of current abundance (which includes both the uncertainty in the most recent count *as well as* the time elapsed since that most recent count). To estimate the abundance in a region encompassing multiple sites, we simply summed the samples from the distribution representing our best estimate for each site to arrive at a distribution for their sum. This procedure allowed us to estimate uncertainty regarding the total population in any region, such as with each of the Small-Scale Management Units. When summing groups of sites, we have propagated the uncertainties inherent to each site’s current abundance by sampling from a truncated (0, $\infty$) Gaussian distribution with the mean equal to the population estimate and with standard error equal to 2.5% (2$\sigma \hspace{0.17em}=\hspace{0.17em}$5% for accuracy = 1), 5% (2$\sigma$ = 10% for accuracy = 2), 12.5% (2$\sigma$ = 25% for accuracy = 3), 25% (2$\sigma$ = 50% for accuracy = 4), and 45% (2$\sigma$ = 90% for accuracy = 5). We opted to use a truncated Gaussian distribution here, rather than the bias-corrected log-normal distribution used by Che-Castaldo et al.^44^, because the extreme skew of the latter distribution for accuracy 5 counts, which are common in the database, interfered with a sensible estimate of the difference between two populations, which was needed to identify which colonies changed significantly in abundance over the past several decades.

### Change in abundance

At each site, we compared our current abundance estimate with a previous population estimate to determine the degree to which populations increased, decreased, or remained stable over time. In most cases, this benchmark population estimate was from the early to mid-1980s, which provided an approximately 40-year span over which to judge population change. To evaluate population change, we drew random samples from the distributions representing the historic count and the updated count, and differenced them to create a distribution of population changes. This procedure allowed us to propagate uncertainties from the population estimates to an estimate for population change. If more than 95% of this distribution indicated either an increase (decrease), we designated that colony as having “increased” (“decreased”), whereas 87.5–95% of the distribution indicating an increase (decrease) would yield the designation “probable increase” (“probable decrease”). If either no updated abundance estimate was available, or if no historic estimate was available, the population change was designated “unknown”.

This population assessment involved no contact with animals, though we report on previously collected but unpublished data obtained under a survey protocol approved by the Stony Brook University Institutional Animal Care and Use Committee (IRBNet ID 237420) and carried out under a permit granted by the National Science Foundation under the Antarctic Conservation Act (45 CFR §673 et seq.) with an initial environmental evaluation approved by the U.S. Environmental Protection Agency Office of Federal Activities.

## Supplementary information

Supplementary Information 1.

Supplementary Information 2.

## Data Availability

All count data generated or analyzed for this study are included with this published article as a Supplementary Information file (1 file in .xlsx format). Supplementary files defining bounding boxes for all penguin sites are also provided (695 files in .dbf, .prj, .qpj, .shp, and .shx formats).
