# Supplementary material for: A global population assessment of the Chinstrap penguin (Pygoscelis antarctica)
Source: Sci Rep. 2020 Nov 10;10:19474. doi: 10.1038/s41598-020-76479-3 (PMC7655846; doi:10.1038/s41598-020-76479-3)
Supplement: Supplementary file 1 — Supplementary Information 1. [file 41598_2020_76479_MOESM1_ESM.pdf]

Supplementary Documents

A global population assessment of the Chinstrap penguin (*Pygoscelis antarctica*)

Noah Strycker[1], Michael Wethington[2], Alex Borowicz[2], Steve Forrest[2], Chandi Witharana[3], Tom Hart[4], and Heather J. Lynch[2,5]

[1]School of Marine and Atmospheric Sciences, Stony Brook University, Stony Brook, New York, USA

[2]Department of Ecology and Evolution, Stony Brook University, Stony Brook, New York, USA

[3]Department of Natural Resources and the Environment, University of Connecticut, Storrs, Connecticut, USA

[4]Department of Zoology, University of Oxford, Oxford, UK

[5]Institute for Advanced Computational Science, Stony Brook University, Stony Brook, New York, USA

Supplementary Figure

Figure S1. Decision tree for assessing Chinstrap penguin colonies.

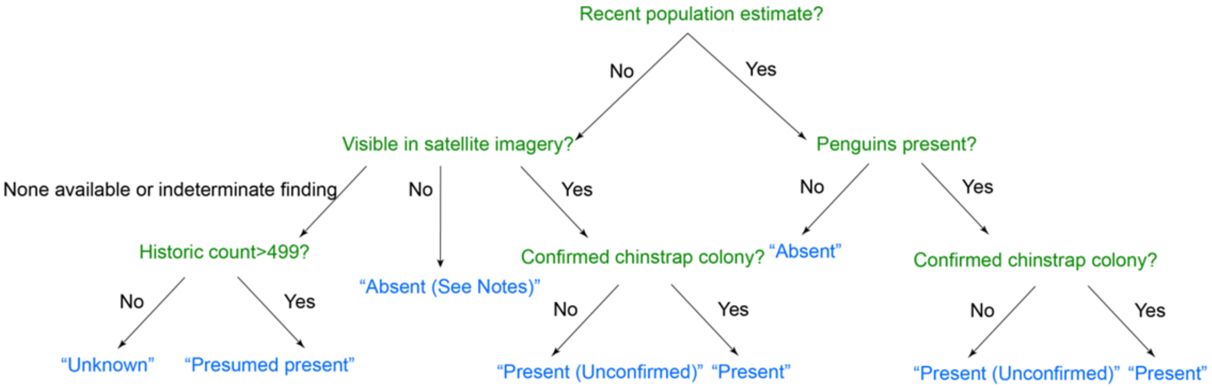

Supplementary Data File

Please note. The data for this manuscript are provided in an Excel spreadsheet.

Supplementary Shape Files

Please note. Files are provided delineating all the bounding boxes we used to define the Chinstrap penguin sites. There are a total of 695 files in .dbf, .prj, .qpj, .shp, and .shx formats.
